# Improving mortality prediction in critically ill cancer patients with a multidimensional machine learning model

**DOI:** 10.1186/s40635-026-00939-9

**Published:** 2026-07-01

**Authors:** Víctor H. Nieto, Adriana C. Aya, Andrés F. Cardona, Edwin Pulido, Heidy Trujillo, Natalia Sánchez, Daniel Molano, Nicolle Wagner-Gutiérrez, Oscar Arrieta, Christian Rolfo, Giovanni Nigita, Joseph Nates

**Affiliations:** 1 Intensive Care Unit, Luis Carlos Sarmiento Angulo Cancer Treatment and Research Center (CTIC), Bogotá, Colombia; 2 Institute of Research and Education, Luis Carlos Sarmiento Angulo Cancer Treatment and Research Center (CTIC), Cra. 14 #169-49, Bogotá, Colombia; 3 GIGA Research Group, Luis Carlos Sarmiento Angulo Cancer Treatment and Research Center (CTIC), Universidad El Bosque, Bogotá, Colombia; 4 Research and Education Direction, Luis Carlos Sarmiento Angulo Cancer Treatment and Research Center (CTIC), Bogotá, Colombia; 5 Gillings School of Global Public Health, University of North Carolina at Chapel Hill, Chapel Hill, USA; 6 General Management, Instituto Nacional de Cancerología - INCAN, Mexico City, Mexico; 7 Division of Medical Oncology, Department of Internal Medicine, The Ohio State University, Comprehensive Cancer Center, The Ohio State University, Columbus, OH USA; 8 Department of Critical Care Medicine, Division of Anesthesiology, Critical Care, and Pain Medicine, The University of Texas MD Anderson Cancer Center, Houston, TX USA

**Keywords:** Prediction model, Machine learning, CatBoost, Cancer patients, Intensive care unit

## Abstract

**Background:**

Prognostic assessment in critically ill cancer patients is challenging due to the suboptimal performance of traditional severity scores. We developed and validated machine learning models to provide an objective triage and risk-stratification tool at admission, identifying patients who require high-intensity organ support.

**Methods:**

We conducted a retrospective cohort study including 997 critically ill cancer patients admitted to the ICU. Forty-six demographic, oncologic, physiological, laboratory, and therapeutic variables collected at ICU admission were used to train and validate ML models. Eight algorithms were evaluated using stratified cross-validation with feature selection and hyperparameter optimization. Model performance was assessed using discrimination, calibration, and classification metrics. Model interpretability was explored using Shapley additive explanations (SHAP).

**Results:**

CatBoost achieved the best performance for ICU mortality prediction (AUROC 0.94), showing excellent discrimination and calibration, and outperforming other ML models. Prediction of 30-day survival was less accurate (best AUROC 0.74), reflecting the influence of post-ICU factors not captured at admission. Key predictors of ICU mortality included severity of organ dysfunction, therapeutic objectives, vasopressor and methylene blue use, SAPS III score, lactate, platelet count, and blood urea nitrogen. For 30-day survival, baseline physiological status, admission type, SAPS III, lactate, creatinine, age, and body mass index were most relevant. SHAP analysis demonstrated that acute physiology and organ dysfunction, rather than cancer diagnosis alone, primarily drove short-term outcomes.

**Conclusions:**

ML-based models, particularly CatBoost, outperformed traditional tools in predicting ICU mortality. Within this oncologic cohort, short-term outcomes were driven primarily by acute physiological derangements rather than specific cancer characteristics. These results support using ML-based risk stratification as an objective triage tool at admission. External validation is warranted to confirm generalizability before clinical integration.

##  Introduction

Critically ill patients with cancer are increasingly admitted to intensive care units (ICUs) as advances in oncologic therapies have improved survival and expanded indications for aggressive supportive care [1–4]. Despite these advances, this population remains particularly vulnerable, with higher risks of infection, organ dysfunction, and short-term mortality compared with non-oncologic ICU patients [5–7]. Accurate prognostic assessment is therefore essential to guide clinical decision-making, allocation of resources, and discussions regarding goals of care [6].

Traditional ICU severity scores, including APACHE, SOFA, and SAPS, are widely used to estimate prognosis; however, their performance in patients with cancer is limited. Validation studies have shown heterogeneous and often inconsistent results, with frequent underestimation or overestimation of mortality in oncologic populations [8, 9]. Even prognostic tools specifically developed for patients with cancer have demonstrated variable accuracy across clinical contexts, tumor types, and stages of disease, underscoring the complexity of integrating malignancy-related factors with acute critical illness [8, 10, 11].

Machine learning (ML) approaches have recently shown promising results in critical care and oncology by integrating multidimensional data and modeling nonlinear relationships. Several ML-based models have improved outcome prediction in general ICU populations and selected oncologic scenarios; however, evidence in critically ill cancer patients remains scarce and largely limited to disease-specific or narrowly defined cohorts [12–17].

Improving prognostic prediction in this population is crucial to reduce bias, support individualized decision-making, and ultimately improve patient outcomes. Accordingly, this study aims to develop and validate machine learning–based models to predict mortality in critically ill cancer patients admitted to the ICU, and to provide an objective risk-stratification tool at admission to support clinical triage and identify patients requiring high-intensity organ support.

## Methods

### Study design and participants

**Fig. 1:**
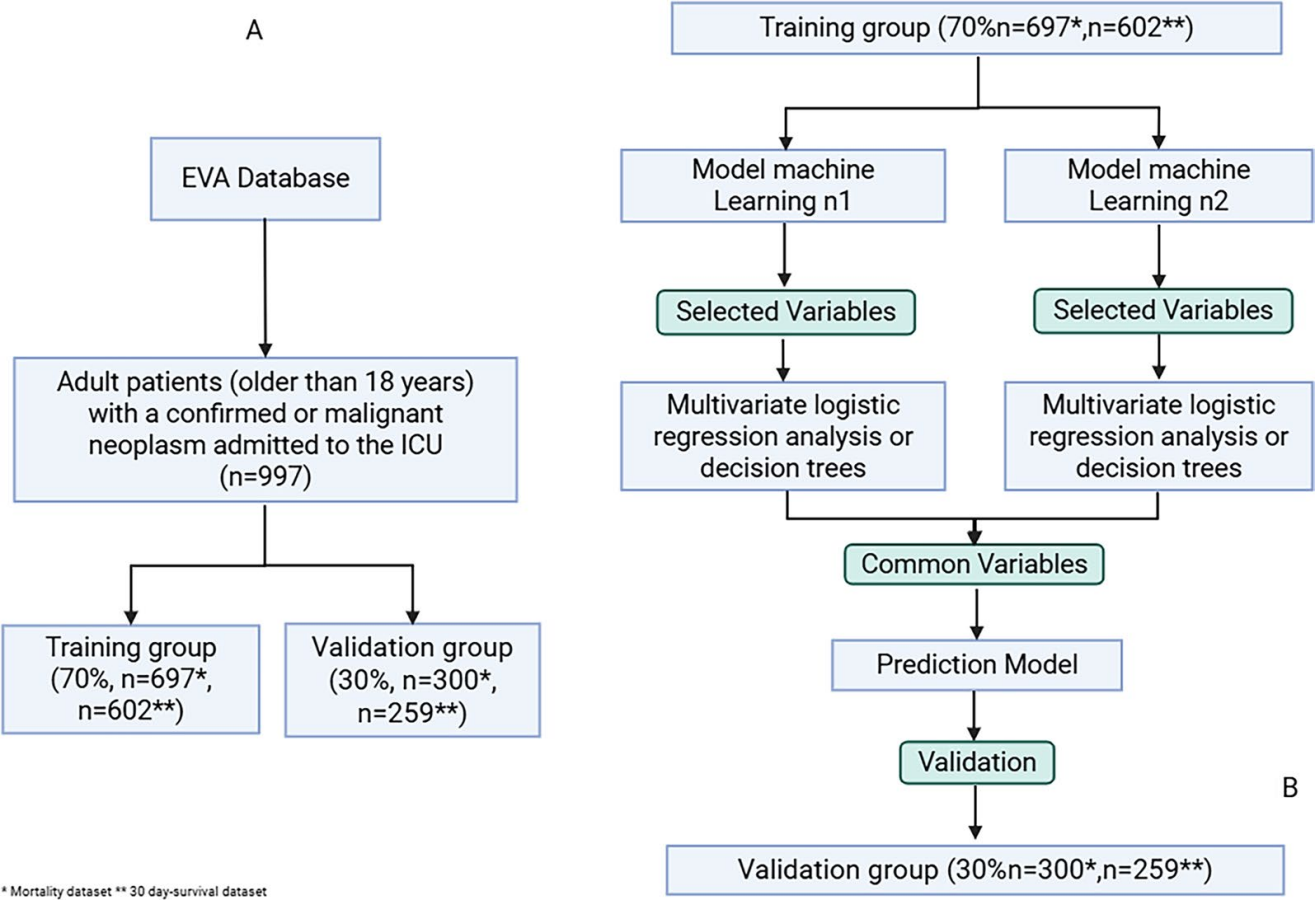
(A) Patient flow diagram. EVA Database; ICU, intensive care unit. (B) Model development flowchart

The dataset was derived from the ambispective EVA (Evidence–Verification–Analysis) cohort at a high-complexity oncology center in Bogotá, Colombia. Approved by the local Ethics Committee (Cayre Approval No. 164 of 2025), the study included 997 adult ICU patients with confirmed or suspected malignancies between July 2022 and July 2025 (Fig. 1 A). To ensure its utility as an early triage tool, all predictors were collected strictly within the first 24 h of admission. Furthermore, feature selection prioritized causal coherence by excluding post-admission outcomes (e.g., length of stay) to prevent temporal leakage. The overall modeling workflow—from preprocessing to performance evaluation—is detailed in Fig. 1B. For the 30-day survival analysis, a sub-cohort of 861 patients was utilized.

## Outcomes and definitions

The primary outcomes were ICU mortality and 30-day survival, both analyzed as binary variables. Candidate predictors were selected based on prior literature identifying prognostic factors in critically ill patients, including those with cancer. A total of 46 variables collected at ICU admission were included. These comprised demographic characteristics (age, sex, body mass index), oncologic variables (primary tumor site, cancer status for solid and hematologic malignancies, performance status, presence of driver mutations, and recent oncologic treatments), physiological and laboratory parameters measured within ± 6 h of ICU admission (including hematologic, renal, metabolic, and coagulation markers), therapeutic interventions (vasopressor use, methylene blue, renal replacement therapy, and mechanical ventilation), and clinical context variables (type of admission, ICU readmission, presence of infection or shock, delirium assessed by CAM-ICU, and severity scores including SAPS III, APACHE II, and SOFA).

## Development of machine learning models

Patients were randomly split into training (70%) and validation (30%) sets. Model robustness and generalizability were ensured using ten-fold stratified cross-validation within the training dataset. Missing continuous variables were imputed within each fold to prevent information leakage, and all features were standardized using z-score normalization. Feature selection was performed using least absolute shrinkage and selection operator (LASSO) regression within each fold to account for collinearity and reduce overfitting. To ensure causal coherence, variables representing post-admission outcomes or subjective clinical intent (e.g., length of stay, therapeutic objective) were strictly excluded from the predictor set.

Two independent prediction models were developed for ICU mortality and 30-day survival using eight machine learning algorithms: Logistic Regression, Random Forest, Extra Trees, Gradient Boosting, AdaBoost, LightGBM, XGBoost, and CatBoost. Hyperparameter optimization was performed within each cross-validation fold. Class imbalance was addressed when applicable, including the use of scale_pos_weight for XGBoost. Model performance was evaluated on the held-out validation sets using discrimination and calibration metrics. Discrimination was assessed via the area under the receiver operating characteristic curve (AUROC). Calibration, the agreement between predicted probabilities and observed outcomes was assessed using calibration plots (reliability curves), the Brier score, and calibration intercept and slope. These metrics were used to determine the clinical reliability of the models’ probability estimates. Classification performance was further evaluated using accuracy, precision, recall, and F1-score. Performance metrics were aggregated across folds to enable a stable comparison.

## Model interpretability

To enhance interpretability of the best-performing models, Shapley additive explanation (SHAP) values were computed. SHAP analysis enabled both global and patient-level assessment of feature contributions, allowing visualization of feature importance, directionality of effects, and individual risk drivers through summary, dependence, and decision plots. This approach facilitated transparent interpretation of complex model outputs in clinically meaningful terms.

### Statistical analysis

Continuous variables are reported as medians with interquartile ranges, and categorical variables as counts and percentages. Comparisons between training and validation datasets were performed using the Wilcoxon rank-sum test for continuous variables and Fisher’s exact test for categorical variables. All statistical analyses and model development were conducted using Python 3.11 with open-source libraries, including Pandas and NumPy for data processing, scikit-learn for machine learning, SciPy for statistical testing, and Matplotlib and Seaborn for visualization. A two-sided p-value < 0.05 was considered statistically significant. The analytical workflow was designed to ensure reproducibility and transparency. The source code is available upon request or via a public GitHub repository:*(*https://github.com/adri1207/Cancer-icu-prediction.git*).*

## Results

### Baseline characteristics

Baseline characteristics provide clinical context in which the machine learning (ML) models were developed and validated. A total of 997 critically ill cancer patients were included in the ICU mortality model, and a subset of 861 patients was analyzed for 30-day survival. Each cohort was randomly divided into training and validation sets (mortality: 697/300; survival: 602/259). Baseline characteristics are summarized in Tables 1 and 2.

**Table 1:** Baseline patient characteristics mortality model

Patients Mortality	All patients *N* = 997	Training dataset *N* = 697	Validation dataset *N* = 300	*P*-value
**Demographic Characteristics**				
Age, years	62 (48–71)	68 (48–70)	62 (49–73)	0.2769
Female	504 (50.6)	341(48.9)	163 (54.3)	0.1287
BMI	23.7 (20.7–26.9)	23.9 (20.6–26.9)	23.4 (20.8–26.6)	0.4224
**Oncological history**				
ECOG performance status				0.1204
*0–2*	848 (85.1)	590 (84.7)	258 (86)	
*3–5*	149 (14.9)	107 (15.3)	42 (14)	
Origin of the tumor				0.5535
*Solid tumor*	787 (78.9)	554 (79.5)	233 (77.7)	
*Hematologic tumor*	210 (21.1)	143 (20.5)	67 (22.3)	
Staging/risk*				0.7222
*Advanced/high*	305 (30.6)	212 (30.4)	93 (31)	
*Early/low*	295 (29.6)	209 (30)	86 (28.7)	
No data	397 (39.7)	276 (39.6)	121 (40.3)	
Sequencing/molecular study or FISH positive**	119 (11.9)	84 (12)	35 (11.7)	0.2747
**Physiological parameters**				
Lactate, mmol/L	1.7 (1.2–2.4)	1.6 (1.2–2.4)	1.7 (1.3–2.5)	0.2046
Excess base	−2.9 (−5.0−0.9)	−2.8 (−4.9−0.9)	−3.0 (−5.4−1.0)	0.3117
Bicarbonate, mmol/L	20.9 (18.8–22.6)	21.0 (18.9–22.6)	20.8 (18.4–22.5)	0.4807
PaFi ratio	258 (214–303.3)	260.4 (215.2–303.3)	253.7 (204.9–303.5)	0.2437
Absolute leukocytes, mm³	10,465 (6392.5–14790)	10,510 (6245–14815)	10,325 (7027.5–14705)	0.7049
Absolute neutrophils, mcL	8390 (4450–12507.5)	8440 (4360–12517.5)	8255 (4797.5–12377.5)	0.9942
Absolute lymphocytes, µL	805.0 (450.0–1367.5)	780(452.5–1317.5)	870 (450–1430)	0.3644
Platelet, µL	231,000(131250–318000)	227,000 (132000–317000)	243,500 (130750–330000)	0.3681
Hemoglobin, g/dL	10.8 (8.9–12.9)	10.8 (8.9–13.0)	10.8 (9.2–12.6)	0.8653
Creatinine, mg/dL	0.8 (0.6–1.1)	0.8 (0.6–1.1)	0.8 (0.6–1.1)	0.2757
BUN, mg/dL	17.1 (13.2–23.8)	16.7 (13.0–23.1)	18.1 (13.5–24.7)	0.1052
Potassium, mmol/L	4.2 (3.8–4.5)	4.2 (3.9–4.5)	4.1 (3.8–4.6)	0.4765
Sodium, mmol/L	137.8 (135.6–140.3)	137.7 (135.4–140.2)	138 (135.9–140.5)	0.2123
Calcium, mg/dL	8.5 (8–8.8)	8.5 (8–8.8)	8.5 (8–8.8)	0.8682
aPTT, s	30.3 (28.5–32.8)	30.5 (28.7–33)	30.1 (28.2–32.5)	0.2776
PT, s	13.6 (12.7–15.5)	13.6 (12.7–15.5)	13.7 (12.6–15.4)	0.4289
Total bilirubin, mg/dL	0.6 (0.4–1.3)	0.6 (0.4–1.3)	0.6 (0.4–1.2)	0.4577
Direct bilirubin, mg/dL	0.3 (0.2–0.8)	0.3 (0.2–0.9)	0.3 (0.2–0.8)	0.9839
Indirect bilirubin, mg/dL	0.3 (0.2–0.4)	0.3 (0.2–0.4)	0.3 (0.2–0.4)	0.2823
ALT, U/L	42.5 (27.8–69.7)	41.4 (27.7–69.1)	43.6 (28.2–72.4)	0.4686
AST, U/L	42.4 (31.5–65.3)	42.1 (30.4–67.4)	44.0 (32.8–63.6)	0.3415
**Clinical assessments and ICU admission context**				
APACHE II	11 (8–15)	11 (8–15)	12 (8–15)	0.5442
SAPS III	43 (32–58)	43 (31–58)	45 (34–57)	0.3135
SOFA	3 (2–4.6)	3 (2–4.8)	3 (2–4)	0.9631
Type of admission				0.7268
*Medical*	575 (57.5)	399 (57.3)	176 (58.7)	
*Elective/emergency surgery*	421 (42.3)	297 (42.7)	124 (41.3)	
Sepsis	288 (28.9)	206 (29.6)	82 (27.3)	0.4938
Shock***	331 (33.2)	246 (35.3)	85 (28.3)	0.0336
Delirium	164 (16.4)	117 (16.8)	47 (15.7)	0.7099
**Therapeutic interventions and support measures**				
Ventilatory support****	302 (30.3)	210 (30.1)	92 (30.7)	0.8807
Painkillers use	732 (73.4)	515 (73.9)	217 (72.3)	0.6393
Vasopressors use	407 (40.8)	289 (41.5)	118 (39.3)	0.5742
Sedatives use	253 (25.4)	176 (25.3)	77 (25.7)	0.9368
Methylene blue use	61 (6.1)	20 (6.7)	41 (5.9)	0.6661
Oncological treatment*****	68 (6.8)	51 (7.3)	17 (5.7)	0.4116
Renal support******	43 (4.3)	22 (3.2)	21 (7)	0.0099
**Other characteristics**				
ICU readmission				0.0961
*1 admission*	443 (52.6)	297 (50.5)	146 (57.3)	
*2 admissions*	240 (28.5)	180 (30.6)	60 (3.5)	
*> 3 admissions*	160 (19)	111 (18.9)	49 (19.2)	

Continuous variables are presented as medians and interquartile ranges (IQRs) and were compared using the Wilcoxon rank sum test. Categorical variables are presented as counts and percentages and were compared using Fisher’s exact test. IQR interquartile range, Pressure of Arterial Oxygen/Fraction of Inspired Oxygen, ALT alanine aminotransferase, AST aspartate aminotransferase, APTT activated partial thromboplastin time, PT partial thromboplastin time, APACHE II Acute Physiology and Chronic Health Evaluation, APS III Acute Physiology Score III, SOFA Sequential Organ Failure Assessment, length of stay in the ICU

*Staging in solid tumors, lymphomas and myelomas early or advanced, Risk in leukemias and gliomas high or low **Solid tumor sequencing study, molecular study in leukemias or FISH in myelomas or lymphomas positive *** If the patient presented with any of the following types of shock: Hypovolemic, Obstructive, Distributive, Cardiogenic **** If the patient required any ventilatory support non-invasive mechanical ventilation, high-flow oxygen therapy, or invasive mechanical ventilation ***** If the patient received any type of oncological treatment, such as immunotherapy, chemotherapy, targeted therapy, hormonal therapy, or radiotherapy ****** If the patient required renal replacement therapy, such as hemodialysis or peritoneal dialysis

**Table 2:** Baseline patient characteristics 30-day survival model

Patients 30-day survival	All patients *N* = 861	Training dataset *N* = 602	Validation dataset *N* = 259	*P*-value
**Demographic characteristics**				
Age, years	62 (48–71)	61 (48–71.8)	63 (50–71)	0.4199
Female	442 (51.3)	311 (51.7)	131 (50.6)	0.8236
BMI	23.7 (20.8–26.9)	23.8 (20.9–27)	23.5 (20.7–26.5)	0.3598
**Oncological history**				
ECOG performance status				0.9801
*0–2*	743 (86.2)	519 (86.2)	224 (86.5)	
*3–5*	118 (13.8)	83 (13.8)	35 (13.5)	
Origin of the tumor				0.2933
*Solid tumor*	701 (81.4)	496 (82.4)	205 (79.2)	
*Hematologic tumor*	160 (18.6)	106 (17.6)	54 (20.8)	
Staging/risk*				0.2515
*Early/low*	267 (31)	195 (32.4)	72 (27.8)	
*Advanced/high*	261 (30.3)	178 (29.6)	83 (32)	
No data	333 (38.7)	229 (38)	104 (40.2)	
Sequencing/molecular study positive**	99 (11.5)	75 (12.5)	24 (9.3)	0.2476
**Physiological parameters**				
Lactate, mmol/L	1.6 (1.2–2.3)	1.6 (1.2–2.3)	1.7 (1.3–2.3)	0.7348
Excess base	−2.8 (−4.7−0.9)	−2.8 (−4.7−0.9)	−2.8 (−4.6−1)	0.8743
Bicarbonate, mmol/L	21 (19–22.6)	21 (19 −22.6)	21 (19–22.5)	0.4934
PaFi ratio	263.6 (223.6–306.8)	261.4 (221.1–303.2)	270 (226.9–319.3)	0.0478
Absolute leukocytes, mm³	10,675 (6905–14840)	10,400 (6925–14850)	11,150 (6840–14750)	0.4502
Absolute neutrophils, mcL	8635 (4910–12585)	8480 (4850–12675)	8930 (5180–12430)	0.5675
Absolute lymphocytes, µL	870.0 (500–1395)	800 (490–1380)	855 (500–1382.5)	0.7294
Platelet, µL	244,000 (159750–327500)	245,000 (159000–320000)	241,000 (161000–334000)	0.843
Hemoglobin, g/dL	11 (9.1–13)	11.1 (9.2–13.1)	10.8 (9–12.9)	0.345
Creatinine, mg/dL	0.8 (0.6–1)	0.8 (0.6–1)	0.8 (0.6–1.1)	0.9627
BUN, mg/dL	16.7 (13–22)	16.7 (12.7–21.7)	16.6 (13.7–23.6)	0.1781
Potassium, mmol/L	4.2 (3.9–4.5)	4.2 (3.9–4.5)	4.2 (3.8–4.5)	0.9753
Sodium, mmol/L	138 (135.6–140.3)	138 (135.6–140.3)	138 (135.6–140.4)	0.9311
Calcium, mg/dL	8.5 (8.1–8.9)	8.6 (8.1–8.9)	8.5 (8–8.8)	0.0884
aPTT, s	30.2 (28.4–32.6)	30 (28.2–32.2)	30.9 (28.8–33)	0.008
PT, s	13.6 (12.6–15.4)	13.5 (12.5–15.2)	13.8 (12.8–15.7)	0.0142
Total bilirubin, mg/dL	0.6 (0.4–1.3)	0.6 (0.4–1.3)	0.6 (0.5–1.3)	0.1942
Direct bilirubin, mg/dL	0.3 (0.2–0.8)	0.3 (0.2–0.8)	0.3 (0.2–0.8)	0.1016
Indirect bilirubin, mg/dL	0.3 (0.2–0.4)	0.3 (0.2–0.4)	0.3 (0.2–0.4)	0.2392
ALT, U/L	42.5 (28.2–69.1)	43 (28.3–69)	41.3 (27.9–70)	0.7944
AST, U/L	41.3 (30.9–63.6)	41.6 (31–63.3)	40.7 (30.3–64.3)	0.9782
**Clinical assessments and ICU admission context**				
APACHE II	11 (8–14)	11 (8–14)	12 (8–15)	0.0261
APS III	41 (31–55)	40 (30–55)	44 (32–55.2)	0.0841
SOFA	3 (2–4)	3 (2–4)	3 (1.2–4)	0.9765
Type of admission				0.3331
*Medical*	447 (52)	306 (50.8)	141 (54.7)	
*Elective/emergency surgery*	413 (48)	296 (49.2)	117 (45.3)	
Sepsis	201 (23.3)	131 (21)	70 (27)	0.096
Shock***	226 (26.2)	77 (29.7)	149 (24.8)	0.1295
Delirium	128 (14.9)	81 (13.5)	47 (18.1)	0.0942
**Therapeutic interventions and support measures**				
Ventilatory support****	177 (20.6)	24 (20.6)	53 (20.5)	1
Painkillers use	602 (69.9)	426 (70.8)	176 (68)	0.4186
Vasopressors use	189 (31.4)	96 (37.1)	285 (33.1)	0.1144
Sedatives use	107 (17.8)	44 (17.0)	151 (17.5)	0.8452
Methylene blue use	9 (1)	5 (0.8)	4 (1.5)	0.4645
Oncological treatment*****	50 (5.8)	37 (6.1)	13 (5)	0.634
Renal support******	24 (2.8)	13 (2.2)	11 (4.2)	0.1125
**Other characteristics**				
ICU readmission				0.9002
*1 admission*	391 (52.9)	274 (52.5)	117 (53.9)	
*2 admissions*	213 (28.8)	153 (29.3)	60 (27.6)	
*> 3 admissions*	135 (18.3)	95 (18.2)	40 (18.4)	

Continuous variables are presented as medians and interquartile ranges (IQRs) and were compared using the Wilcoxon rank sum test. Categorical variables are presented as counts and percentages and were compared using Fisher’s exact test. IQR interquartile range, Pressure of Arterial Oxygen/Fraction of Inspired Oxygen, ALT alanine aminotransferase, AST aspartate aminotransferase, APTT activated partial thromboplastin time, PT partial thromboplastin time, APACHE II Acute Physiology and Chronic Health Evaluation, APS III Acute Physiology Score III, SOFA Sequential Organ Failure Assessment, length of stay in the ICU

*Staging in solid tumors, lymphomas and myelomas early or advanced, Risk in leukemias and gliomas high or low **Solid tumor sequencing study, molecular study in leukemias or FISH in myelomas or lymphomas positive *** If the patient presented with any of the following types of shock: Hypovolemic, Obstructive, Distributive, Cardiogenic **** If the patient required any ventilatory support non-invasive mechanical ventilation, high-flow oxygen therapy, or invasive mechanical ventilation ***** If the patient received any type of oncological treatment, such as immunotherapy, chemotherapy, targeted therapy, hormonal therapy, or radiotherapy ****** If the patient required renal replacement therapy, such as hemodialysis or peritoneal dialysis

In the ICU mortality cohort, the median age was 62 years (IQR, 48–71), and 50.6% of patients were female. The median APACHE II score at admission was 11 (IQR, 8–15), and the median 24-hour SOFA score was 3 (IQR, 2–4.6). Minor differences between the training and validation sets were observed for the presence of shock (*p* = 0.0336) and renal replacement therapy (*p* = 0.0099), while all other variables were well balanced.

In the 30-day survival cohort, baseline age and severity scores were comparable between the training and validation sets (APACHE II: 11 [IQR, 8–14]; SOFA: 3 [IQR, 2–4]), and 52% of patients had solid tumors. Statistically significant differences were noted for the PaO₂/FiO₂ ratio, partial thromboplastin time, and APACHE II score (all *p* < 0.05), although overall clinical characteristics remained largely comparable. Taken together, both cohorts demonstrated adequate balance between training and validation subsets, supporting the appropriateness of the random data split for model development.

## Development and assessment of ML models

**Fig. 2:**
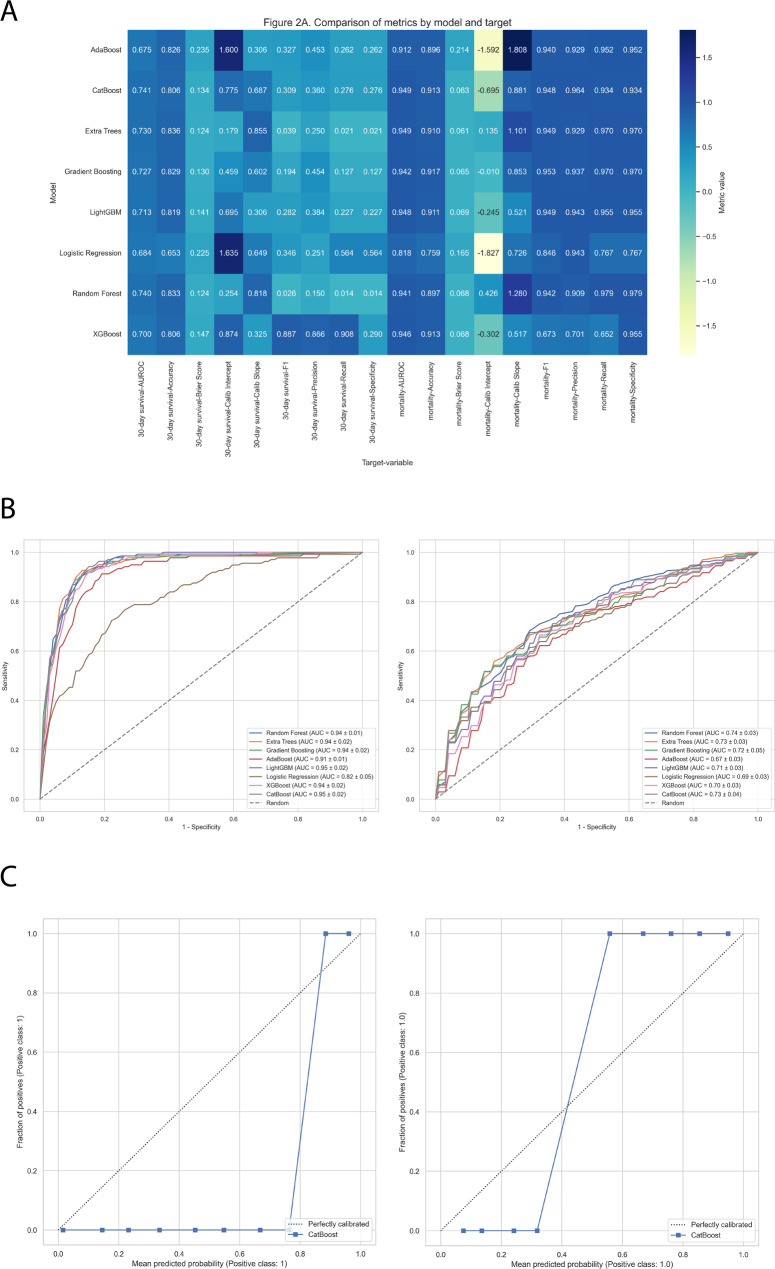
A. Comparisson of metrices by model and target. **B**. ROC curves mortality; ROC curves 30-day survival. **C**. Calibration plots for mortality; Calibration plots for 30-day survival

Among the evaluated algorithms, CatBoost achieved the superior performance for ICU mortality prediction, yielding an AUROC of 0.949, accuracy of 91.3%, and an F1-score of 0.948. Beyond discrimination, the model demonstrated excellent reliability; calibration analysis (Fig. 2 A C) revealed a Brier score of 0.063 and a calibration slope of 0.88, indicating that predicted probabilities highly correspond to observed clinical outcomes. While LightGBM showed comparable discriminative power (AUROC 0.948), traditional Logistic Regression performed significantly lower (AUROC 0.818) and exhibited poorer calibration metrics. Full performance metrics for all models are summarized in Fig. 2 A and B.

Predicting 30-day survival proved more complex, likely due to the influence of post-ICU clinical factors. Nonetheless, CatBoost maintained the most balanced performance with an AUROC of 0.741 and a Brier score of 0.134. Although Random Forest and Extra Trees reached similar AUROC values (0.740 and 0.730, respectively), CatBoost offered a more robust calibration profile (Fig. 2 A C), with a calibration slope of 0.69. AdaBoost consistently showed the lowest performance (AUROC 0.675), with limited sensitivity for identifying long-term survivors. Detailed comparative metrics for this outcome are presented in Fig. 2 A and B.

### Key predictors and model interpretability

**Fig. 3:**
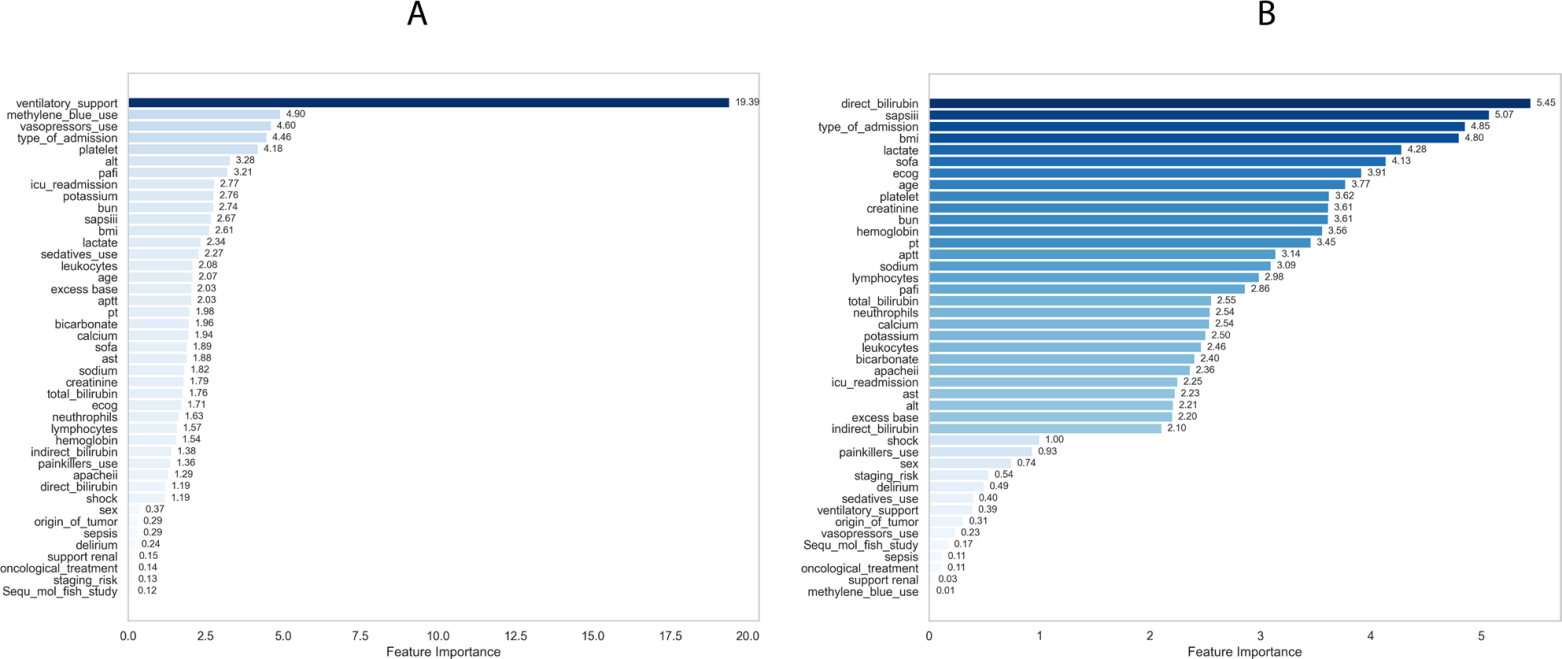
A. Feature importance-CatBoost mortality model. **B**. Feature importance-CatBoost 30-Day Survival Model

Feature importance analysis identified distinct patterns for ICU mortality and 30-day survival prediction. For ICU mortality, ventilatory support emerged as the most influential predictor, followed by therapeutic objectives, methylene blue use, vasopressor support, ICU length of stay, and admission type. Key laboratory and severity markers, including platelet count, blood urea nitrogen, SAPS III score, and lactate level, also contributed substantially to model predictions (Fig. 3 A).

In the 30-day survival model, SAPS III was the most important predictor, followed by direct bilirubin, body mass index, admission type, lactate, sodium, age, and other baseline clinical variables. In contrast to ICU mortality, cancer-related characteristics and pre-ICU physiological status were more influential than acute ICU interventions (Fig. 3B).

**Fig. 4:**
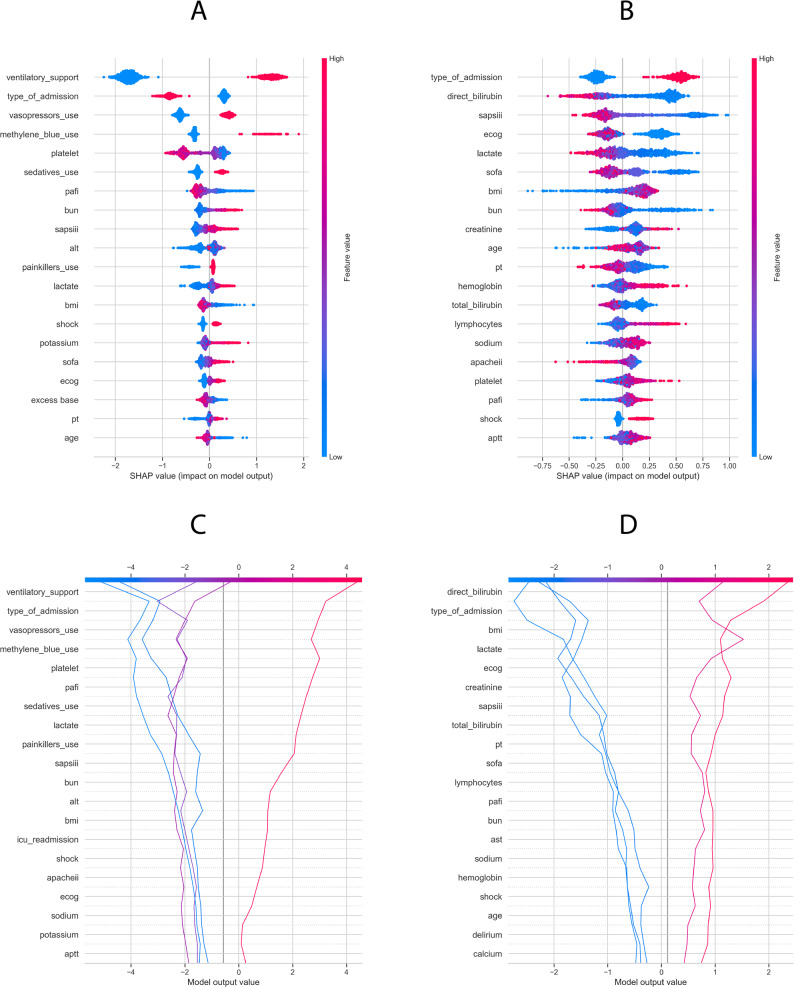
A. SHAP Summary plot-mortality model. **B**. SHAP Summary plot 30-day survival model. **C**. SHAP Decision plot-mortality model. **D**. SHAP Decision plot 30-day survival model

To enhance interpretability, SHAP analysis was applied to the best-performing models. SHAP summary plots (Fig. 4 A and B) illustrate the global importance of features and the direction of their effects on predicted outcomes. For ICU mortality, the strongest contributions were related to disease stage, therapeutic objectives, organ dysfunction, and acute ICU support. For 30-day survival, predictions were primarily driven by baseline patient characteristics and physiological reserve.

SHAP dependence plots highlighted nonlinear relationships and interactions between variables, such as the modifying effect of vasopressor use or disease stage on the association between lactate levels and mortality risk. SHAP decision plots (Fig. 4 C and D) provided patient-level explanations, demonstrating how individual features contributed cumulatively to final risk predictions. Overall, SHAP analysis reinforced the clinical relevance of organ dysfunction, baseline condition, and treatment intensity, while supporting transparent and individualized risk stratification.

## Discussion

In this study, we developed and validated machine learning (ML) models to predict ICU mortality and 30-day survival in a large cohort of critically ill cancer patients. Among the evaluated algorithms, CatBoost demonstrated excellent discrimination for ICU mortality and the most balanced performance for 30-day survival. The models consistently showed that short-term outcomes were primarily driven by acute physiology, severity of organ dysfunction, and treatment intensity rather than by cancer diagnosis alone.

The intended application of this CatBoost model is to serve as an objective triage and risk-stratification tool during the first 24 h of admission. By providing an early mortality estimate based on acute physiological derangements rather than subjective intent-of-care, the model can support clinicians in identifying patients who may benefit from aggressive therapeutic escalation. Nevertheless, to avoid self-fulfilling prophecies, the model’s output should be integrated as a complementary data point within a broader clinical decision-making framework, rather than a standalone determinant of treatment limitation.

These findings highlight the complex interaction between baseline patient condition, acute critical illness, and therapeutic context in determining outcomes in oncologic ICU patients. The predominance of variables related to cardiovascular instability, tissue hypoperfusion, and organ failure such as vasopressor and methylene blue use, SAPS III score, lactate, platelet count, and renal function suggests that short-term mortality in this population is largely driven by the severity of acute physiological derangements superimposed on the underlying oncologic condition, rather than by cancer diagnosis alone [18, 19]. Accurate early risk stratification may therefore support more timely and tailored interventions, including not only the initiation but also the appropriate intensity of organ support and therapeutic escalation. Moreover, the ability to estimate risk before ICU admission could facilitate earlier referral and admission, potentially improving outcomes by avoiding delayed access to advanced critical care [20].

In contrast, 30-day survival was more strongly influenced by baseline physiological reserve and chronic patient factors, including admission type, nutritional status, bilirubin, creatinine, and age. These findings indicate that longer-term outcomes are partly determined by pre-existing vulnerabilities and organ dysfunction present at ICU admission, many of which may be less modifiable in the acute setting [21, 22]. The lower predictive performance observed for 30-day survival likely reflects the contribution of post-ICU factors such as tumor progression, oncologic treatment decisions after discharge, and limitations in therapeutic effort that are not captured by admission-based models and inherently constrain longer-term prediction.

Previous prognostic models in oncology populations have largely relied on traditional regression-based approaches. Karaboyun et al. proposed a mortality prediction model for advanced-stage cancer patients requiring unplanned hospitalization during systemic therapy; however, this model was not designed for ICU patients and lacked external validation [10]. Similarly, a systematic review by Cabrera Losada et al. demonstrated that conventional ICU severity scores, including APACHE II, SAPS III, and SOFA, frequently under- or overestimated mortality in patients with cancer, underscoring the limitations of traditional tools in this population [8]. In the general critically ill population, ML models have shown incremental improvements over standard severity scores. Nikravangolsefid et al. reported modest but consistent improvements in mortality prediction using machine-learning approaches, particularly logistic regression and eXtreme Gradient Boosting, in large cohorts of patients with sepsis [12], Likewise, Iwase et al. demonstrated high predictive accuracy using random forest models in large, unselected ICU populations, including a cohort of over 12,000 patients, and identified lactate dehydrogenase (LDH) as a novel prognostic biomarker [13].

Within oncologic ICUs, machine-learning applications remain limited, disease-specific, and fragmented in nature. Studies focusing on selected conditions—such as lung cancer, hyperkalemia, or immunotherapy-related toxicity—have demonstrated promising predictive performance but lack generalizability across heterogeneous cancer ICU populations. For example, Huang et al. developed several ML algorithms to predict in-hospital mortality in critically ill lung cancer patients, achieving strong discrimination (AUC 0.92) and identifying SOFA score, albumin, and bilirubin as key predictors [14]. Similarly, Bu et al. proposed an interpretable ML model for patients with cancer and hyperkalemia, integrating SHAP and LIME to identify urine output and heart rate as novel prognostic variables [15]. Other work, such as that by Wang et al., has explored ML-based prediction of immunotherapy-related toxicity [23]. Although these studies highlight the potential value of ML in oncologic critical care, their narrow clinical scope limits applicability to broader and more diverse cancer ICU populations.

From a methodological perspective, this study applied a rigorous and reproducible modeling framework with robust data handling. To ensure causal coherence and avoid temporal leakage, variables that are determined concurrently with the outcome or that reflect post-admission clinical decisions such as length of stay and therapeutic objective were strictly excluded from the feature set. This ensures that the model relies exclusively on information available at the moment of prediction, providing a truly independent signal of patient risk. Missing data were imputed using median-based and KNN strategies within the cross-validation folds to prevent information leakage. Feature selection was performed using LASSO within each fold, accounting for collinearity while preserving clinically relevant predictors and reducing overfitting. Model training incorporated stratified cross-validation and algorithm-specific hyperparameter tuning. Furthermore, to move beyond simple discriminative performance, we conducted a comprehensive calibration assessment. This is critical for clinical utility, as it ensures that the predicted probabilities of mortality closely align with the observed event rates, a prerequisite for any tool intended to support bedside decisions [24, 25].

Several strengths and limitations should be considered. Strengths include the large real-world cohort and the use of explainable AI. However, our findings are subject to case-mix dependency, as the model was developed in a single high-complexity oncology ICU in Bogotá. The specific prevalence of certain malignancies and the local standard of care may influence feature importance and model calibration. Therefore, external validation across diverse centers and healthcare systems is not just a recommendation but a strict prerequisite to ensure transportability and to mitigate the risk of clinical bias before any bedside deployment.

In conclusion, ML-based models, particularly CatBoost, demonstrated superior performance in predicting ICU mortality compared to traditional prognostic tools. Within this oncologic cohort, short-term outcomes were primarily driven by acute physiological derangements and the severity of organ dysfunction rather than specific cancer-related characteristics. These findings support the potential of ML-based risk stratification as an objective triage tool at ICU admission. External validation is warranted to confirm the transportability of the model and to support its future integration into clinical decision-making frameworks in oncologic critical care.

## Data Availability

The datasets supporting the conclusions of this article are publicly available in the PhysioNet repository: https://physionet.org/projects/dmq4yv6S96QvyTE5Ex3b/overview/. The code used for data preprocessing, model development, and statistical analysis is publicly available in the GitHub repository: https://github.com/adri1207/Cancer-icu-prediction. Access to the dataset is subject to PhysioNet credentialing requirements and compliance with the applicable data use agreements.
